# Context aware decision support in neurosurgical oncology based on an efficient classification of endomicroscopic data

**DOI:** 10.1007/s11548-018-1806-7

**Published:** 2018-06-13

**Authors:** Yachun Li, Patra Charalampaki, Yong Liu, Guang-Zhong Yang, Stamatia Giannarou

**Affiliations:** 1 The Hamlyn Centre for Robotic Surgery, Imperial College London, London, UK; 2 Institute of Cyber-Systems and Control, Zhejiang University, Hangzhou, China; 3 Department of Neurosurgery, Cologne Medical Center, University Witten, Cologne, Germany; 4 Department of Neurosurgery, Heinrich Heine University Duesseldorf, Duesseldorf, Germany

**Keywords:** Brain tissue characterisation, Image and video classification, Deep learning

## Abstract

**Purpose:**

Probe-based confocal laser endomicroscopy (pCLE) enables in vivo, in situ tissue characterisation without changes in the surgical setting and simplifies the oncological surgical workflow. The potential of this technique in identifying residual cancer tissue and improving resection rates of brain tumours has been recently verified in pilot studies. The interpretation of endomicroscopic information is challenging, particularly for surgeons who do not themselves routinely review histopathology. Also, the diagnosis can be examiner-dependent, leading to considerable inter-observer variability. Therefore, automatic tissue characterisation with pCLE would support the surgeon in establishing diagnosis as well as guide robot-assisted intervention procedures.

**Methods:**

The aim of this work is to propose a deep learning-based framework for brain tissue characterisation for context aware diagnosis support in neurosurgical oncology. An efficient representation of the context information of pCLE data is presented by exploring state-of-the-art CNN models with different tuning configurations. A novel video classification framework based on the combination of convolutional layers with long-range temporal recursion has been proposed to estimate the probability of each tumour class. The video classification accuracy is compared for different network architectures and data representation and video segmentation methods.

**Results:**

We demonstrate the application of the proposed deep learning framework to classify Glioblastoma and Meningioma brain tumours based on endomicroscopic data. Results show significant improvement of our proposed image classification framework over state-of-the-art feature-based methods. The use of video data further improves the classification performance, achieving accuracy equal to 99.49%.

**Conclusions:**

This work demonstrates that deep learning can provide an efficient representation of pCLE data and accurately classify Glioblastoma and Meningioma tumours. The performance evaluation analysis shows the potential clinical value of the technique.

## Introduction

Biophotonics techniques such as probe-based confocal laser endomicroscopy (pCLE) have enabled direct visualisation of tissue at a microscopic level, with recent pilot studies suggesting it has a clear role in identifying residual cancer tissue and improving resection rates of brain tumours [16]. However, the interpretation of endomicroscopic information remains challenging, particularly for surgeons who do not themselves routinely review histopathology. Also, even among experts, the diagnosis can be examiner-dependent, leading to considerable inter-observer variability. Automatic tissue characterisation with pCLE, based on a database of previously annotated data by expert physicians with diagnosis confirmed by histology, would support the surgeon in establishing diagnosis and could also guide autonomous robotic tissue scanning to focus locally on pathological areas.

State-of-the-art computer-aided diagnosis (CAD) systems designed for the analysis of pCLE data have mainly focused on the classification of colonic cancers. A content-based image and video retrieval framework based on the Bag-of-visual Words (BoW) approach has been proposed in [2] for the differentiation of neoplastic and benign colorectal polyps. A local dense multiscale image descriptor has been introduced to extract a set of features which are clustered into a number of “visual words", whose frequencies build the image signature. The similarity between two images can be estimated by calculating the distance between their signatures. This image retrieval method has been extended for video retrieval by relying on the coarse registration of images taken at different times. Clinical evaluation of this framework presented in [1] shows that automated classification of pCLE videos of colonic polyps achieves high performance, comparable to off-line diagnosis of pCLE videos established by expert endoscopists. The above video retrieval framework has been extended in [3] to include high-level knowledge for the pathological interpretation of pCLE images. For that purpose, binary semantic concepts commonly used by expert endoscopists to diagnose pCLE videos of colonic polyps have been extracted and used as additional information that complements the visual outputs of the retrieval framework.

Recently, approaches to content-based image retrieval of pCLE data have focused on learning discriminative visual features to improve the retrieval accuracy. In [10], a Multi-View Multi-Modal Embedding (MVMME) framework has been proposed to learn discriminative features of pCLE videos by exploiting both mosaics and histology images. Visual features were extracted from the pCLE mosaics using multiple operators such as SIFT and HOG and a mapping from these features to a latent space was learned in a supervised way to generate robust latent features which are more discriminative than unimodal features from mosaics alone. This work has been extended in [9] where an unsupervised multimodal graph mining (UMGM) approach has been proposed to learn the discriminative features for pCLE mosaics of breast tissue. A multiscale multimodal graph is built based on pCLE mosaics and histology patches, and a latent feature space is created to learn discriminative features without supervision.

Wan et al. [22] extended the content-based image retrieval framework proposed in [2] using an efficient feature encoding scheme based on codeword proximity. A set of keypoints has been uniformly sampled from the pCLE images, and different state-of-the-art descriptors such as SIFT and FREAK have been used to describe the keypoints. The keypoint descriptors are encoded with the proposed linear locality constraint (LLC) scheme which exploits spatial locality of the visual features instead of the hard quantization employed in BoW-based approaches. The above framework was applied for the characterisation of brain tissue. A support vector machine (SVM) classifier was used to classify from pCLE images two types of commonly diagnosed brain tumours, namely Glioblastomas and Meningiomas. This work has been extended in [12] by exploring more encoding schemes for data description and using a majority voting-based classification scheme for video classification.

The above CAD systems are composed of three main steps, namely visual feature extraction, feature encoding, and supervised classification. The performance of these systems relies on the design of handcrafted morphological or textural descriptors, capable of extracting discriminative image features which could facilitate the classification task. However, the selection of efficient features is problem-dependent and it is not easy to find a generic feature extraction method which can perform equally well in different applications. Hence, the above CAD systems can have significant performance variations depending on the data and the surgical context.

Recently, significant progress has been made in image feature extraction and representation, mainly due to the revival of deep learning models such as convolutional neural networks (CNN). These architectures enable the learning of data-driven and highly representative image features from a large set of training data. This alleviates the need to design explicit handcrafted image features and also allows the feature extraction, encoding and classification tasks to be done within the optimisation of the same deep architecture. Hence, the network performance can be easily tuned, in a systematic fashion.

The success of deep learning models in general computer vision tasks has motivated their application to medical image analysis [20]. More specifically, they have been used to detect and segment organs from multiple imaging modalities [4, 6], semantic segmentation in histological images [23], multimodal registration, image super-resolution, image classification [15] and workflow analysis [21]. Deep learning has also gained a lot of attention for the design of CAD systems, focusing on abnormality detection and tissue state classification [5, 17]. CNN models have been extensively employed for this purpose and extract image features either by training the models from scratch or using transfer learning when a limited number of training data is available. Transfer learning has shown promising performance and extracts image features using off-the-shelf CNNs pre-trained on different data (such as natural images) or performing pre-training of the models on big datasets and then supervised fine-tuning on medical data.

The aim of this paper is to propose a brain tissue characterisation framework for context aware decision support in neurosurgical oncology. This will be part of a platform which can extract, interpret and use context information to provide the surgeon with a set of clinical actions that can be made in the current situation and guide intraoperative resection. The context in our case refers to the discriminative information which is extracted from the pCLE data and is used to characterise the state of the tissue. In particular, we focus on the classification of brain tumours into Glioblastoma and Meningioma which are the most common malignant and benign tumours, respectively, in neurosurgery. Our classification is based on the analysis of endomicroscopic data, and we propose two deep learning-based frameworks for the classification of image and video pCLE data. To the best of our knowledge, this is the first work on brain tissue characterisation based on deep learning. The novel aspects of our work include:

- An efficient representation of the context information of pCLE images by exploring state-of-the-art CNN models and proposing different tuning configurations. The best configurations have been selected and used to classify image and video data into the above tumour types.
- A novel video classification framework based on the combination of convolutional layers with long-range temporal recursion to estimate the probability of each tumour class.
- A set of network architectures and video segmentation methods which has been combined with different pCLE data representations to increase the video classification accuracy.

The proposed framework has been validated on ex vivo pCLE data and has been compared to state-of-the-art brain tumour classification approaches. The performance evaluation analysis shows the potential clinical value of the proposed framework.

## Methods

In this work, we propose two deep learning-based frameworks for brain tissue characterisation based on the classification of image and video pCLE data into two tumour types, namely Glioblastoma and Meningioma.

### Image classification

Our image-based tissue characterisation approach employs convolutional neural networks for context representation and classification of pCLE images. A CNN for classification can be thought of as the composition of a number of convolutional functions $f(\mathbf x )=f_L(\ldots f_2(f_1(\mathbf x ; \mathbf w _1);\mathbf w _2) \ldots ;\mathbf w _L)$, where *L* is the number of layers, $\mathbf x$ is an input image and $\mathbf w =[\mathbf w _1,\ldots ,\mathbf w _L]$ are the network parameters learned during training. The convolutional operations are applied using a sliding window over the image to detect generic features. The convolutions are followed by a nonlinear function, the Rectified Linear Unit (ReLU), applied to each element of the convolution output. The feature map extracted at each layer becomes input for the next layer. In order to reduce the dimensionality of the extracted features, the maps from each convolution are downsampled through a max-pooling layer which keeps the maximum value of the features in the pool. After the convolution and pooling layers, fully connected layers are introduced where each pixel is considered as a separate neuron just like a regular neural network. The final fully connected layer has the same number of outputs as the number of labels in the classification task and those values represent the likelihood of each label, estimated using the softmax function.

**Fig. 1:**
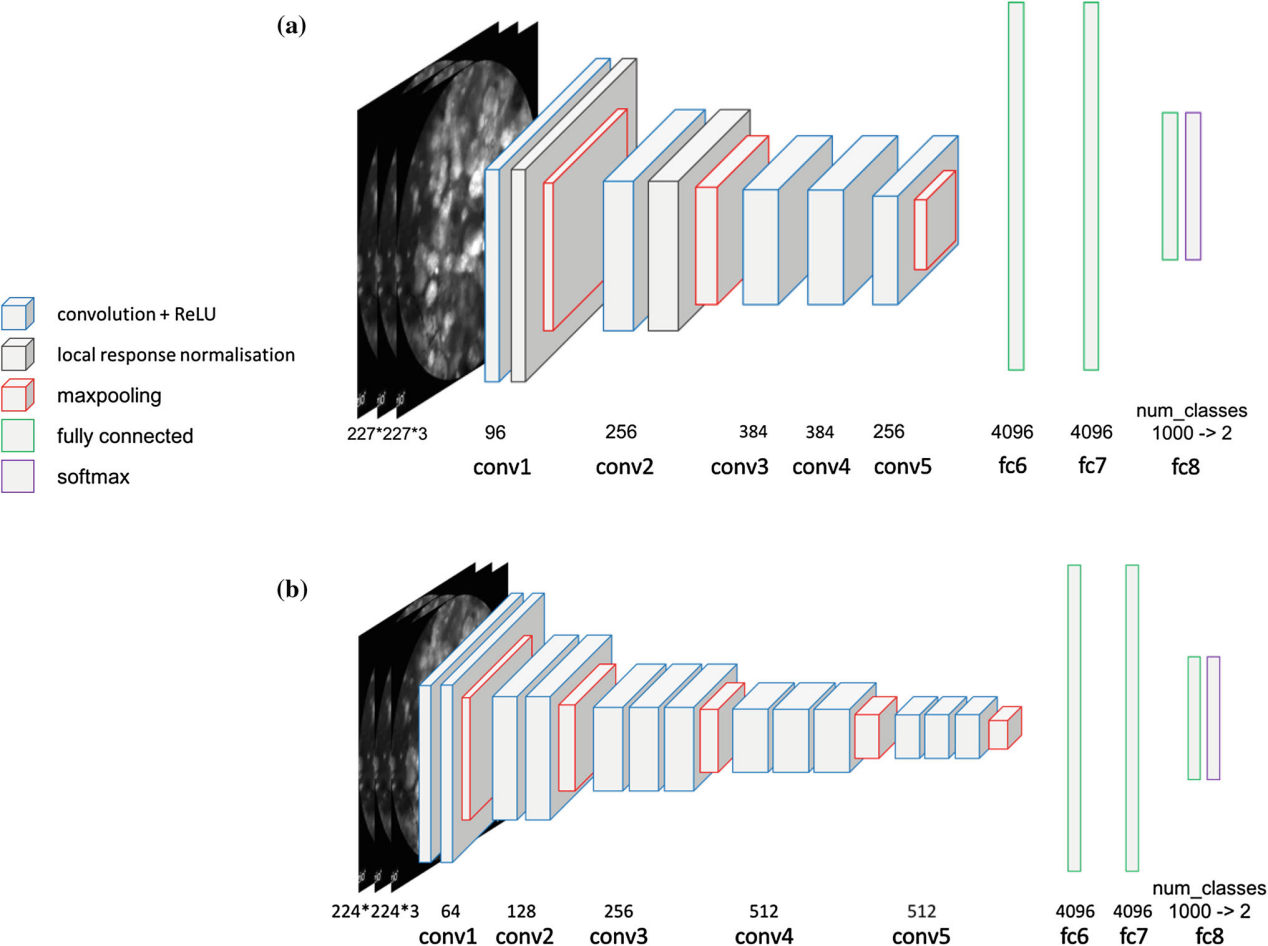
Layer clustering in the employed CNN architectures. **a** AlexNet, **b** VGG16

In this work we employ three state-of-the-art CNN architectures namely, the AlexNet [14], the VGG16 [18] and the Inception-v3 [19] to classify two types of brain tumour. The AlexNet is an eight-layer CNN which is composed of 5 convolutional layers followed by 3 fully connected layers. The VGG16 network is much deeper, consisting of 13 convolutional layers and 3 fully connected layers. We cluster the convolutional layers into 5 groups which are separated by max-pooling layers as shown in Fig. 1. In the rest of our analysis, we denote the convolutional layer groups as conv1, conv2, $\ldots$, conv5 and the fully connected layers as fc6, fc7, fc8. The Inception is a fully convolutional network including various Inception modules with parallel structure. Different to AlexNet and VGG16, which have 3 fully connected layers at the end, the Inception network has only one fully connected layer combined with a softmax layer. As we only need to differentiate two types of tumour, we change the output of the last layer in the above networks from 1000 to 2. Also, since these networks require colour images as input and the endomicroscopic data in our dataset is greyscale, we simply copy the grey values to the second and third channel to produce 3-channel input images.

Upon initialization, a CNN is simply a function which through numerous operations transforms highly dimensional data into several numerical values which at this point are random. It is through training that the model function $z=f(\mathbf x ;\mathbf w )$ learns to achieve a desired goal. More specifically, during training the parameters $\mathbf w$ are learned by evaluating the performance of the network using a loss function which in the case of classification is directly related to the probabilities allocated to the correct classes. In this work, in order to efficiently represent the endomicroscopic data, we choose not to train our CNNs from scratch or fine-tune all the layers. Instead, we propose two approaches to tune the above CNN architectures.

In the first approach, called *feature extractor*, we randomly initialise the weights and biases of several of the 8 layer groups and then train those variables as usual. More specifically, we tune the first $(8-n),~(n=1,~2,~3,~ \ldots ,~8)$ layers using parameters from pre-trained models and do not update them during training. The rest *n* layers are initialised randomly, with the weights sampled from Gaussian distributions and the biases set to a constant value. Another approach is the *fine-tuning*, where we initialise the weights and biases of some trainable layers from pre-trained models and then fine-tune those values using a smaller learning rate. Similar to the above tuning approach, we copy to the first $(8-n)$ layers the weights and biases of the employed CNN models pre-trained on the ImageNet dataset and do not update them during training. For the later *n* layers, we transfer the pre-trained values and update them at a learning rate equal to the 1/10th of the pre-training rate [8] as pre-training has already provided good initialisation for those layers. The parameter *n* is here referred as the tuning depth. Since the Inception network has a different architecture, we retrain only the final fully connected layer which corresponds to tuning depth $n=1$.

**Fig. 2:**
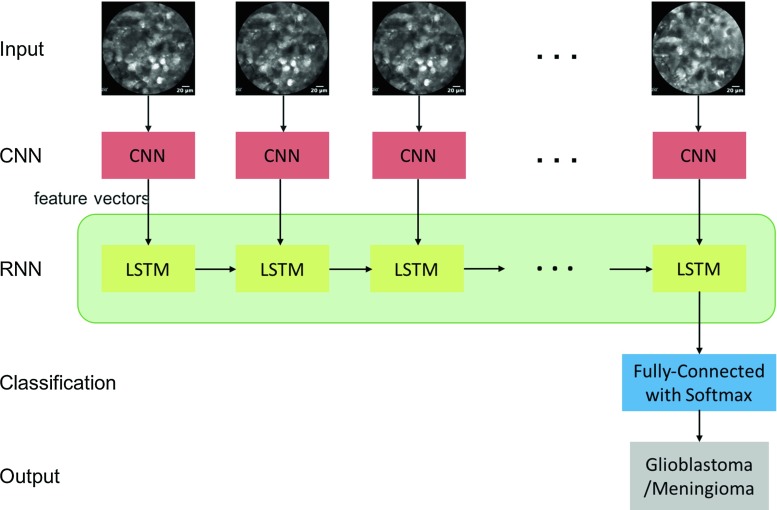
Overview of proposed approach for video classification. The LSTM in the green box may contain more than one LSTM layers

For the last layer of both architectures, we cannot use the values from pre-trained models since *fc*8 has been modified to fit our binary classification task. So, the last layer is always randomly initialised in both approaches. Training the CNN from scratch is a special case of the *feature extractor* method where we optimise all the layers. The case of fine-tuning all the layers is covered when we use the *fine-tuning* approach with tuning depth $n=8$.

### Video classification

For the classification of video pCLE data, we use a deep learning architecture which combines convolutional layers and long-range temporal recursion. Specifically, we extract visual features from a set of consecutive endomicroscopic images using a CNN network and then feed the ordered feature sequences to a recurrent neural network (RNN) to perform classification. At the end of the network, a fully connected layer with softmax is included to generate a prediction about the tumour class. An outline of the proposed architecture is shown in Fig. 2.

**Fig. 3:**
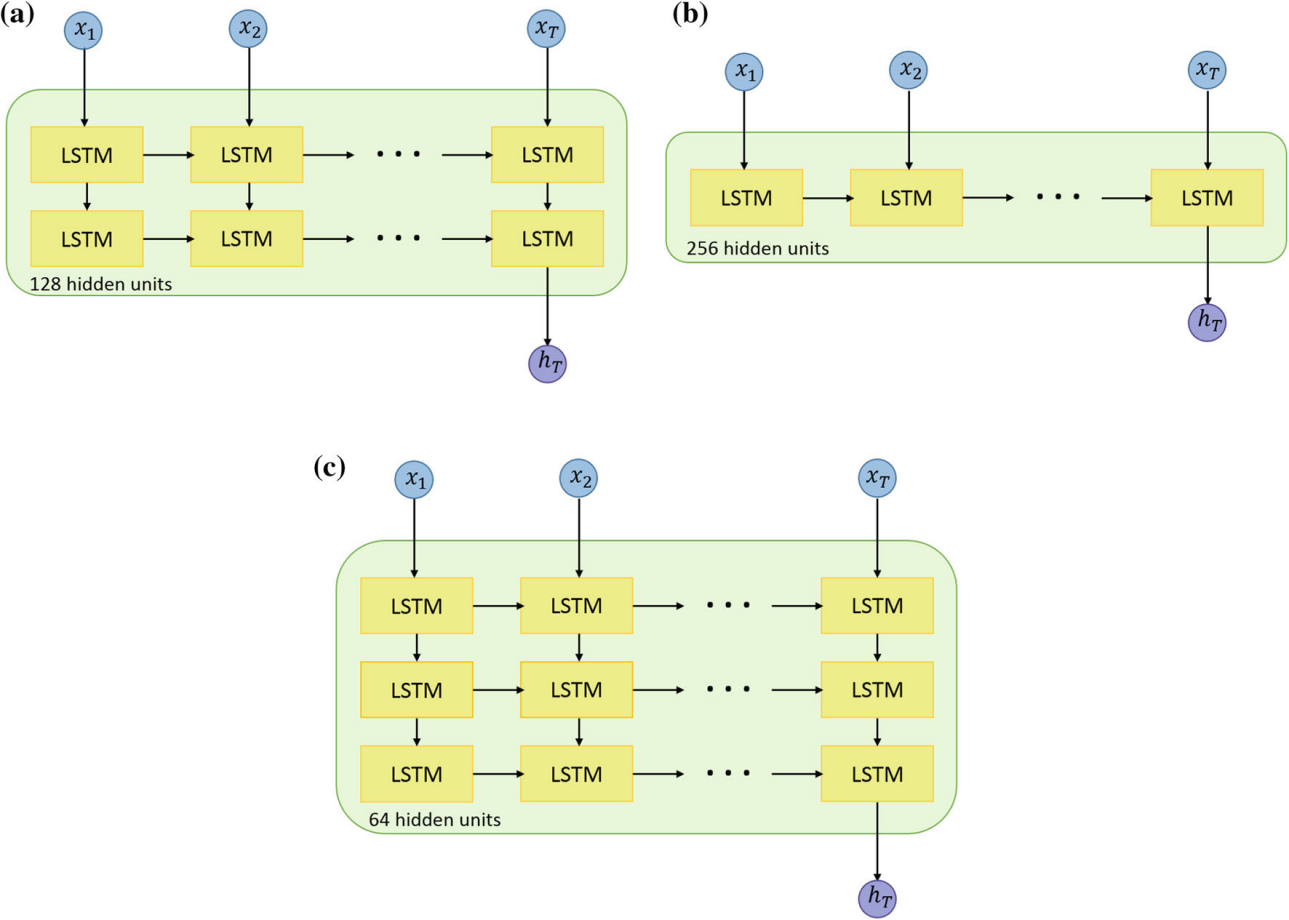
LSTM architectures with different width and depth

To extract visual features from the pCLE images, we employed the same CNN models that were used for image classification. For temporal recursion, we use the long short-term memory (LSTM) network [11]. We explore 3 different architectures with different width (number of hidden units) and depth (number of LSTM layers), namely the *Standard LSTM* with 2 LSTM layers, the *Wide LSTM* with only one but wider layer and the *Deep LSTM* of 3 layers as shown in Fig. 3.

In this work, we test two different configurations to combine the CNN and the LSTM networks, namely the *Before-Final-fc* (*BFF*) and *After-Final-fc)* (*AFF*). In the former configuration, we pass to the LSTM network the input of the final fully connected layer. This is a feature vector of length 2048, 4096, 4096 for the Inception network, the AlexNet and the VGGNet, respectively. In the latter configuration, the input of the LSTM network is the output of the last fully connected with softmax layer of the CNN network which is a vector of size equal to the number of classes in the classification task and represents the predictions of the classes.

The above architecture is different to the long-term recurrent convolutional networks proposed in [7] as our visual feature extraction is not based on the hybrid CaffeNet and ZFNet model. Also, the LSTM configuration in [7] includes only one layer with different number of hidden units. In our framework, we train the CNN and LSTM networks separately rather than training the architecture end-to-end which provides a more efficient training and allows us to train the LSTM with various features. Finally, our final prediction is based on the probability at time step T while [7] is using the average of all label probabilities from time step 1 to T.

## Experiments

In this section, we first describe the preprocessing steps that we apply on our endomicroscopic data. Then we evaluate the performance of the proposed image and video classification methods.

### Data preprocessing

**Fig. 4:**
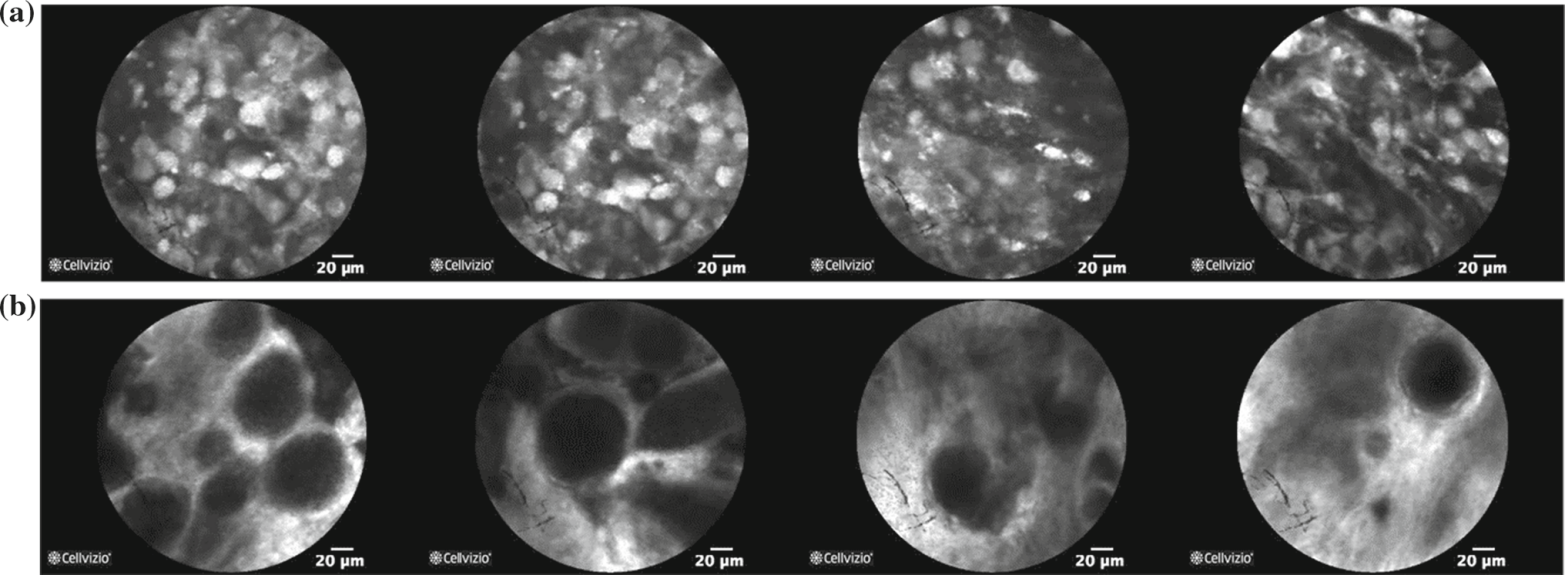
Sample images of our pCLE dataset. **a** Glioblastoma, **b** Meningioma

The validation of the proposed classification framework is based on ex vivo pCLE data of Meningioma and Glioblastoma brain tumours, collected during brain tumour resection procedures at the Merheim Hospital in Germany. The examined tissue samples were scanned in the surgical theatre next to the patient, immediately after removing the tumour. As contrast agent, acriflavine hydrochloride AF from Sigma Pharmaceuticals, Victoria, Australia, was used. The data were collected using the Cellvizio system (Mauna Kea Technologies, Paris, France) and were classified into the above two types by expert histopathologists. Each pCLE video represents one tumour type and corresponds to a different patient. Our dataset is a subset^1^ of the dataset used in [22] and includes 16 videos for Glioblastoma and 17 for Meningioma, with 25,000 frames in total (13,862 for Glioblastoma and 11,616 for Meningioma). Sample frames of our data are shown in Fig. 4. The frames of our dataset are rectangular images of size $464 \times 336$. As the employed CNNs require square images as input, we first centrally crop the pCLE images into images of size $336 \times 336$ by removing the black area on their left and right site and then resize them into the appropriate size for each network ($227 \times 227$ for AlexNet, $224 \times 224$ for VGGNet, and $299 \times 299$ for Inception).

**Fig. 5:**
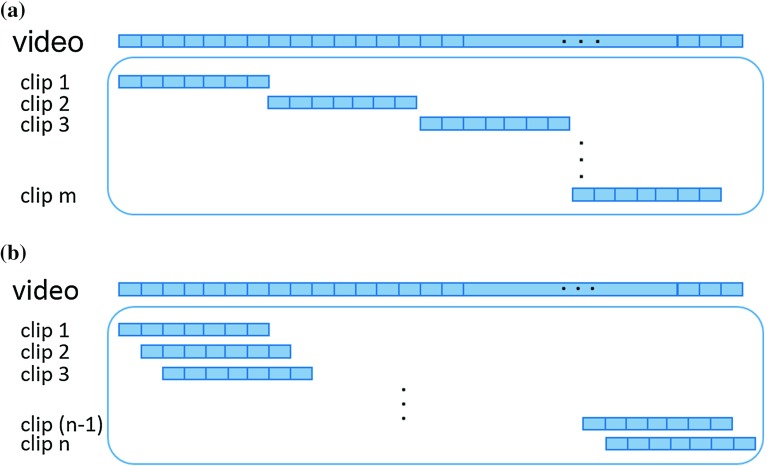
Video dataset expansion. In **a** $m=L / k$ and in **b** $n=L-(k-1)$, where *k* is the length of the video clip and *L* the length of the video sequence. **a** Video segmentation with non-overlapping video clips, **b** video segmentation with overlapping video clips

For the video classification approach, to expand the dataset for both training and validation, we segment the videos into short video clips. The proposed video classification framework is tested using overlapping and non-overlapping video clips. In the non-overlapping case, no frame appears in two video clips at the same time as shown in Fig. 5a. Overlapping video clips are generated as shown in Fig. 5b by shifting the frame selection block by one frame at a time. In both types of clipping, we ensure that no video clip contains frames from two different videos.

To evaluate the performance of the different models and configurations proposed in this work, we split the dataset into 3 parts, namely the training set (66%), the validation set (17%) and the test set (17%). The training set is used to fit the parameters of each model while the validation set to compare their performance and decide which model to select. The performance of the selected models is then assessed on the test set. To ensure independence of each set, we perform patient-level (i.e. video-level) splitting and no frame from one set ends up on the other sets. To eliminate data splitting bias we employ sixfold cross-validation, where the test and validation sets are selected such that they are different for each run, while the rest of the dataset is used for training. The size of the test set for each fold during image and video classification is shown in Table 1. The test set size for each fold changes slightly because of our video-level splitting. All experiments report average classification performance on the sixfold.

**Table 1:** Test set size, in number of frames and clips, for each cross-validation fold during image and video classification, respectively

Method	Fold 1	Fold 2	Fold 3	Fold 4	Fold 5	Fold 6	Total
Image classification	4384	4500	4228	4552	3812	4002	25,478
Video classification							
Overlapping	4149	4265	4040	4270	3530	3673	23,927
Non-overlapping	88	92	86	93	78	80	517

**Table 2:** Image classification cross-validation results on the validation and test sets

Dataset	Configuration	Accuracy	TPR	FPR	Precision	$F_1$ measure
Validation	Alex *extract* $n=5$	**0.9813**	**0.9857**	**0.0216**	**0.9732**	**0.9790**
	Alex *tune* $n=5$	0.9783	0.9836	0.0253	0.9687	0.9757
	Alex *tune* $n=3$	0.9791	0.9810	0.0221	0.9728	0.9766
	VGG *tune* $n=5$	0.9830	0.9875	0.0200	0.9748	0.9807
	VGG *tune* $n=3$	**0.9831**	**0.9812**	**0.0145**	**0.9815**	**0.9811**
	Inception *tune* $n=1$	**0.9631**	**0.9730**	**0.0440**	**0.9481**	**0.9598**
Test	Alex *extract* $n=5$	0.9797	0.9824	0.0218	0.9735	0.9775
	VGG *tune* $n=3$	0.9851	0.9803	0.0106	0.9871	0.9834
	Inception *tune* $n=1$	0.9661	0.9718	0.0375	0.9552	0.9629

Bold value indicates the best models with the highest accuracy

**Fig. 6:**
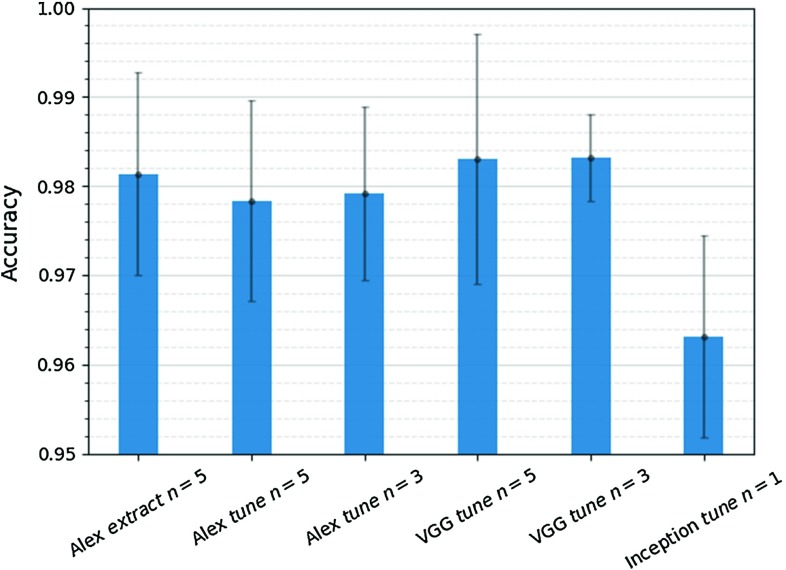
Image classification accuracy of sixfold cross-validation on the validation set. Error bars represent the 95% confidence interval of mean accuracy

### Image classification

The hyperparameters we apply when training our models are those used for AlexNet in [14]. The AlexNet and VGG16 models are trained for 1000 iterations (approximately 10 epochs depending on the size of the training set), using gradient descent with mini-batch size 128. The learning rate is fixed to 0.01 for the *feature extraction* method, while for the *fine-tuning* it is 10 times smaller, i.e. 0.001. Our network minimises the cross-entropy loss function during the training optimisation process. For the Inception network, we set the number of training iterations to 4000. The last layer is retrained using the gradient descent optimiser with learning rate 0.01, batch size 100 and cross-entropy as the loss function. We use the TensorFlow framework to train our models.

To constrain the set of classification models for validation, we first assess the performance of all possible combinations of tuning methods and tuning depths proposed in “Image classification” section without cross-validation, to select the best classifiers. We refer to tuning depths $n=1,2$ as shallow tuning, $n=3,4,5,6$ as medium tuning and $n=7,8$ as deep tuning. The models with the best performance were the (a) **Alex** ***extract*** $n=5$, (b) **Alex** ***tune*** $n=5$, (c) **Alex** ***tune*** $n=3$, (d) **VGG** ***tune*** $n=5$, (e) **VGG** ***tune*** $n=3$. These models together with the **Inception** ***tune*** $n=1$, were selected for further performance evaluation with sixfold cross-validation.

The performance of the selected models on the validation and test datasets is shown in Table 2, and their classification accuracy is compared in Fig. 6. From the above results, it can be seen that the best configuration for the AlexNet is the *feature extractor* with tuning from conv4 to fc8, achieving 97.97% classification accuracy on the test set. For the VGGNet, the *fine-tuning* with tuning from fc6 to fc8 is the best with 98.51% classification accuracy on the test set. The Inception network has the lowest accuracy, equal to 96.61%. It is also clear from Fig. 6 that the **VGG** ***tune*** $n=3$, with the highest accuracy and lowest variance, outperforms the other models.

In general, as we increase the depth of tuning *n*, the features tend to be more low-level. The above results indicate that model tuning of medium depth can generate discriminative features and provide an efficient representation of our pCLE data. This enables the selected classifiers to recognise the delicate difference between the two tumour types and generate accurate predictions. In our classification task, deep tuning results in lower classification accuracy possibly due to the relatively small size of our training dataset. In the above analysis, we observe that the AlexNet works better with the *feature extractor* tuning method while the VGGNet achieves more accurate results using the *fine-tuning* method. This shows that loading pre-trained weights helps the convergence of deeper and complex networks. Since pCLE data are very different from the dataset used for pre-training, random initialisation can generate efficient data representation when used with simple networks such as the AlexNet.

### Video classification

To extract visual features for video classification, we selected the CNN models that performed the best in the image classification task, namely the **Alex** ***extract*** $n=5$, the **VGG** ***tune*** $n=3$ and the **Inception** ***tune*** $n=1$. To train the LSTM model, we set the batch size to 32 and use the Adam method [13] for optimisation with the default values proposed in [13]. The training epoch is 4 for the overlapping video clip case and 80 for the non-overlapping case. The cross-entropy is still used as our loss function.

**Fig. 7:**
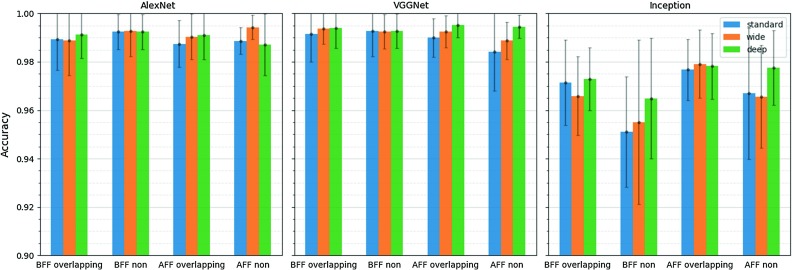
Video classification accuracy of sixfold cross-validation on the validation set. Error bars represent the 95% confidence interval of mean accuracy

For evaluation, we use fixed-length video clips, 48 frames long and report the accuracy of classifying video clips rather than entire videos. The different CNN-LSTM combination approaches (BFF and AFF), video segmentation methods (overlapping and non-overlapping video clips) and LSTM configurations (*Standard LSTM*, *Wide LSTM*, *Deep LSTM*) result in 36 models which are evaluated on the validation dataset and compared in Fig. 7. Detailed performance evaluation results are presented in Table 3. As it can be observed, all the models are able to achieve accurate predictions with classification rate higher than 95%. The configuration ***AFF*** **overlapping** seems to perform the best and for the VGGNet model with **deep** LSTM achieves 99.49% accuracy on the test dataset. The superior performance of our video classification framework verifies that temporal information assists the classification of pCLE data.

**Table 3:** Video classification cross-validation results on the validation and test set

CNN	Dataset	Configurations	Video classification		
			Standard	Wide	Deep
AlexNet	Validation	BFF overlapping	0.9893	0.9887	0.9912
		BFF non-overlapping	0.9924	0.9928	0.9924
		AFF overlapping	0.9875	0.9903	0.9911
		AFF non-overlapping	0.9886	**0.9943**	0.9871
	Test	AFF non-overlapping	0.9866	0.9828	0.9888
VGGNet	Validation	BFF overlapping	0.9916	0.9937	0.9939
		BFF non-overlapping	0.9927	0.9926	0.9927
		AFF overlapping	0.9899	0.9925	**0.9952**
		AFF non-overlapping	0.9842	0.9888	0.9945
	Test	AFF overlapping	0.9918	0.9949	0.9949
Inception	Validation	BFF overlapping	0.9715	0.9659	0.9729
		BFF non-overlapping	0.9510	0.9550	0.9648
		AFF overlapping	0.9768	**0.9791**	0.9782
		AFF non-overlapping	0.9669	0.9656	0.9776
	Test	AFF overlapping	0.9782	0.9858	0.9836

Bold value indicates the best models with the highest accuracy

From the performance evaluation results in Table 3, it can be deduced that the *AFF* configuration outperforms the *BFF*. This is because in the *BFF* configuration the RNN has to learn what the fully connected layers at the end of the CNN are already doing, namely mapping the feature vector to a prediction. On the other hand, the *AFF* directly inputs to the LSTM of the probabilities of two classes and then the LSTM is trained to learn the connections between the different frames. Since the CNN already obtains very accurate results through prior training, the LSTM based on *AFF* further leverages the predictions from the neural network and achieves very good classification accuracy. The different video-clip generation types achieve very similar performance which indicates that extending the size of the training dataset with overlapping clips does not improve the classification accuracy significantly. As for the architecture of the LSTM, wide architectures are able to achieve slightly greater performance in most situations verifying that they are better at sequence learning.

**Fig. 8:**
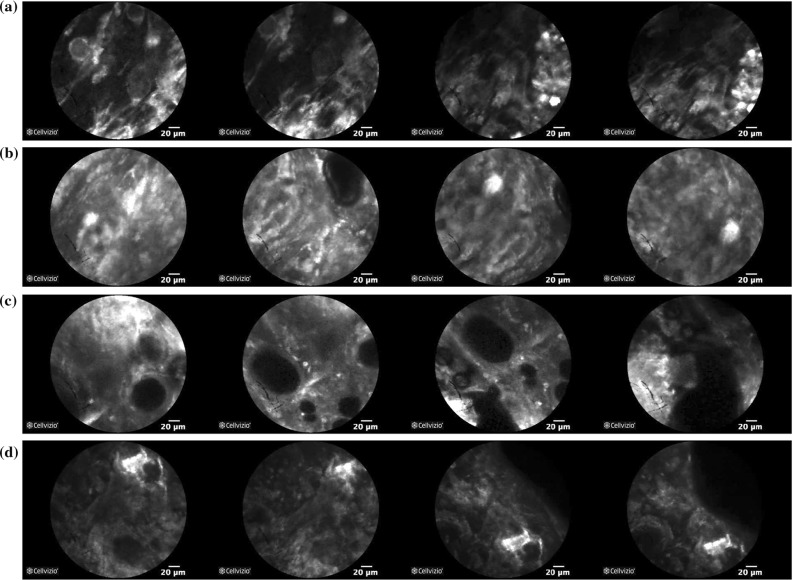
Qualitative results from our video classification using **VGG** ***tune*** $n=3$ for feature extraction, *AFF* combination, overlapping segmentation and deep LSTM. **a** True negative. True label: Glioblastoma, prediction: Glioblastoma. **b** False positive. True label: Glioblastoma, prediction: Meningioma. **c** True positive. True label: Meningioma, prediction: Meningioma. **d** False negative. True label: Meningioma, prediction: Glioblastoma

The qualitative performance evaluation results in Fig. 8 for **VGG** ***tune*** $n=3$, the best video classification configuration, show that the proposed framework can correctly classify challenging data such as the frames in Fig. 8a, c which are not very similar to the typical tumour class data. However, when the appearance of the data differs significantly from the typical tumour class appearance, such as in Fig. 8b or when the data quality is impaired as in Fig. 8d, the proposed video classification fails.

**Table 4:** Classification accuracy of score averaging and majority voting of the image classification results on the original dataset

Dataset	Configurations	Score averaging			Majority voting		
		Inception	AlexNet	VGGNet	Inception	AlexNet	VGGNet
Validation	Overlapping	0.9790	0.9901	0.9948	0.9780	0.9902	0.9939
	Non-overlapping	0.9775	0.9910	0.9945	0.9775	0.9910	0.9945
Test	Overlapping	0.9853	0.9928	0.9952	0.9838	0.9927	0.9943
	Non-overlapping	0.9873	0.9923	0.9964	0.9873	0.9923	0.9964

The performance of our proposed video classification framework has also been compared to score averaging and majority voting of the image classification results on the same temporal window for the non-overlapping and overlapping dataset segmentation methods. Score averaging is done by averaging the output of the softmax layer for each frame on the temporal window and using this average for class prediction. Majority voting refers to averaging over the class predictions for each frame on the temporal window.

As it is shown in Table 4, for video sequences which represent a single tumour class, these approaches can achieve high classification accuracy, comparable to our proposed video classification. However, in practice, during tissue scanning the pCLE video data might contain frames from multiple tissue classes (for instance, healthy tissue and tumour). Since our dataset does not include healthy tissue pCLE data, to simulate the above scenario, we generated using our original dataset, video sequences containing frames from both tumour classes and evaluated the classification accuracy of the above three approaches.

**Table 5:** Classification accuracy of score averaging and majority voting of the image classification results on the mixed video sequences

Dataset	Percent	Configurations	Regular replace					Group replace				
			LSTM			Majority voting	Score averaging	LSTM			Majority voting	Score averaging
			Standard	Wide	Deep			Standard	Wide	Deep		
Validation	2/12	BFF overlapping	0.9905	0.9896	0.9891	0.9921	0.9908	0.9878	0.9889	0.9873	0.9910	0.9909
		AFF overlapping	0.9891	0.9882	**0.9911**			0.9751	**0.9902**	0.9887		
		BFF non-overlapping	0.9873	**1.0000**	0.9873	0.9909	0.9909	0.9963	**1.0000**	0.9945	0.9927	0.9927
		AFF non-overlapping	0.9866	0.9945	0.9945			0.9891	0.9904	0.9945		
	4/12	BFF overlapping	0.9773	0.9792	0.9772	0.9808	0.9795	0.9738	0.9737	0.9736	0.9819	0.9796
		AFF overlapping	0.9782	**0.9825**	0.9793			0.9777	0.9807	**0.9815**		
		BFF non-overlapping	0.9945	0.9982	0.9852	0.9815	0.9815	1.0000	**1.0000**	1.0000	0.9830	0.9830
		AFF non-overlapping	0.9870	**1.0000**	0.9870			0.9891	0.9924	0.9924		
	5/12	BFF overlapping	0.9735	0.9613	0.9595	0.9673	0.9646	0.9637	0.9585	0.8693	0.9654	0.9619
		AFF overlapping	**0.9762**	0.9469	0.9563			**0.9749**	0.9696	0.9247		
		BFF non-overlapping	0.9910	**1.0000**	0.9716	0.9641	0.9641	1.0000	**1.0000**	1.0000	0.9639	0.9639
		AFF non-overlapping	0.9889	0.9964	0.9815			0.9908	0.9924	0.9924		
Test	2/12	AFF overlapping	0.9912	0.9934	0.9917	0.9917	0.9927	0.9770	0.9927	0.9926	0.9920	0.9929
		BFF non-overlapping	0.9853	0.9982	0.9907	0.9927	0.9927	0.9982	1.0000	0.9944	0.9945	0.9945
	4/12	AFF overlapping	0.9834	0.9868	0.9841	0.9824	0.9832	0.9835	0.9859	0.9851	0.9833	0.9840
		BFF non-overlapping						1.0000	1.0000	1.0000	0.9870	0.9870
		AFF non-overlapping	0.9848	1.0000	0.9908	0.9848	0.9848					
	5/12	AFF overlapping	0.9744	0.9429	0.9621	0.9699	0.9644	0.9709	0.9675	0.9273	0.9648	0.9575
		BFF non-overlapping	0.9889	1.0000	0.9830	0.9639	0.9639	1.0000	1.0000	1.0000	0.9582	0.9582

Bold value indicates the best models with the highest accuracy

The mixed video sequences were created following two different frame mixing rules, namely regular replacement and group replacement. In the former case, a mixed video sequence is generated by replacing, in the original sequence in regular intervals, a number of frames with random frames of the opposite tumour class. In the latter case, a group of consecutive frames is replaced considering temporal windows of the same size as our video clips. In addition, different percentage of replaced frames were considered. For example, a video clip with regular replacement of $(2/12)*100\%$ Meningioma (MEN) frames on a Glioblastoma (GBM) sequence would have the following structure [10GBM, 2MEN, 10GBM, 2MEN, 10GBM, 2MEN, 10GBM, 2MEN], while the group replacement would result in [40GBM, 8MEN]. By considering overlapping video clips in our performance evaluation, we introduce randomness in the distribution of the replaced frames in the examined temporal window.

The superiority of our proposed video classification approach compared to score averaging and majority voting of the image classification results is shown in Table 5. The improvement in the classification accuracy provided by our framework is more significant when the percentage of replaced frames is high. Also, from the above performance evaluation study it can be deduced that the frame mixing rule does not affect the classification performance significantly. The performance of our proposed video classification is higher when non-overlapping video clips are used for video segmentation. This is because in that case the LSTM learns the temporal pattern of the replaced frames which is consistent among the datasets. This also explains the good performance of the BFF configuration in the non-overlapping case which is comparable to the AFF configuration.

From the above performance evaluation study, we can deduce that our deep learning-based frameworks can provide an efficient representation of the pCLE data and accurately classify Glioblastoma and Meningioma tumours. The proposed image classification approach with 98.51% classification accuracy outperforms the feature-based brain tumour classification approach presented in [22] with close to 90% accuracy and in [12] with 84.32%. The combination of the VGGNet and LSTM networks for video classification achieves improved performance with classification accuracy equal to 99.49%. These results verify the potential of deep learning-based methods in classifying pCLE data, with significant gain in classification accuracy.

The average running time of our image classification on a GTX 1080 Ti GPU for the AlexNet, the VGG16 and the Inception network is 3.5, 9 and 25 msec, respectively. Our video classification based on pre-computed features (e.g. AFF with VGG) runs at about 1 s.

## Conclusions

In this work, we have proposed a deep learning-based brain tissue characterisation framework which can classify brain tumours into Glioblastoma and Meningioma based on the analysis of pCLE image and video data. For image classification, an efficient representation of the context information of pCLE images has been proposed by exploring different CNN models and tuning configurations. Our video classification is based on a novel combination of convolutional layers with long-range temporal recursion to estimate the probability of each tumour class. The proposed deep learning-based image classification approach with 98.51% classification accuracy outperforms state-of-the-art feature-based brain tumour classification methods. The use of video data further improves the classification performance with accuracy equal to 99.49%. These results verify the potential of deep learning-based methods in classifying pCLE data, with significant gain in classification accuracy. The focus of our future work is to build up a decision support platform which can characterise different states of brain tissue. For this purpose, more brain tissue classes will be examined with pCLE data collected during intraoperative tissue scanning using a contrast agent which has been approved for use in humans.

## Footnotes

1 Due to data ownership reasons, only a subset of the data used in [22] was made available to us and was used for our experimental results.
